# Development and Validation of a Homemade, Low-Cost Laparoscopic Simulator for Resident Surgeons (LABOT)

**DOI:** 10.3390/ijerph17010323

**Published:** 2020-01-02

**Authors:** Domenico Soriero, Giulia Atzori, Fabio Barra, Davide Pertile, Andrea Massobrio, Luigi Conti, Dario Gusmini, Lorenzo Epis, Maurizio Gallo, Filippo Banchini, Patrizio Capelli, Veronica Penza, Stefano Scabini

**Affiliations:** 1 OU Oncological Surgery, IRCCS Ospedale Policlinico San Martino, 16132 Genoa, Italy; soriero.domenico@gmail.com (D.S.); davide_pertile@libero.it (D.P.); massobrioandrea@gmail.com (A.M.); loreepis@gmail.com (L.E.); stefanoscabini@libero.it (S.S.); 2 Department of Surgical Sciences and Integrated Methodologies, University of Genoa, 16132 Genoa, Italy; giulia.atzori90@libero.it; 3 Academic Unit of Obstetrics and Gynecology, IRCCS Ospedale Policlinico San Martino, 16132 Genoa, Italy; 4 UOC General, Vascular and Thoracic Surgery, G. Da Saliceto Hospital, AUSL, 29121 Piacenza, Italy; dr.luigiconti@gmail.com (L.C.); filippobanchini@virgilio.it (F.B.); p.capelli@ausl.pc.it (P.C.); 5 Association of Architects of Bergamo, 24100 Bergamo, Italy; 6 Department of Internal Medicine (Di.M.I.), University of Genoa, 16132 Genoa, Italy; maurizio.gallo@unige.it; 7 Biomedical Robotics Lab, Advanced Robotics Department, Istituto Italiano di Tecnologia, 16152 Genoa, Italy; veronica.penza@iit.it

**Keywords:** laparoscopy, low-cost simulator, homemade simulator, surgical simulation, trainee, students, education

## Abstract

Several studies have demonstrated that training with a laparoscopic simulator improves laparoscopic technical skills. We describe how to build a homemade, low-cost laparoscopic training simulator (LABOT) and its validation as a training instrument. First, sixty surgeons filled out a survey characterized by 12 closed-answer questions about realism, ergonomics, and usefulness for surgical training (global scores ranged from 1—very insufficient to 5—very good). The results of the questionnaires showed a mean (±SD) rating score of 4.18 ± 0.65 for all users. Then, 15 students (group S) and 15 residents (group R) completed 3 different tasks (T1, T2, T3), which were repeated twice to evaluate the execution time and the number of users’ procedural errors. For T1, the R group had a lower mean execution time and a lower rate of procedural errors than the S group; for T2, the R and S groups had a similar mean execution time, but the R group had a lower rate of errors; and for T3, the R and S groups had a similar mean execution time and rate of errors. On a second attempt, all the participants tended to improve their results in doing these surgical tasks; nevertheless, after subgroup analysis of the T1 results, the S group had a better improvement of both parameters. Our laparoscopic simulator is simple to build, low-cost, easy to use, and seems to be a suitable resource for improving laparoscopic skills. In the future, further studies should evaluate the potential of this laparoscopic box on long-term surgical training with more complex tasks and simulation attempts.

## 1. Introduction

Laparoscopic surgery is the gold standard approach for performing many surgical procedures. There are several advantages regarding performing minimally invasive abdominal surgery, such as the reduction of blood transfusions, postoperative pain, and hospitalization time; better aesthetic results; and a quicker return to premorbid functional activity [1]. However, the technical skills required for this approach are different from those needed for open surgery: in particular, laparoscopic surgery involves enhanced hand-eye coordination and the ability to operate while receiving a 2D visual image [2]. Furthermore, laparoscopic instruments are fixed to the abdominal skin, thus constraining the range of operative movements. Moreover, due to a fulcrum effect, they tend to amplify the users’ procedural tremors. For all these reasons, laparoscopic surgery is associated with a significant and variable learning curve, which is different for each specific surgical procedure. Therefore, the early training of laparoscopic skills in medical education has a key role in providing potential benefits for learning minimally invasive abdominal surgery [3,4]. 

Simulation offers the opportunity to practice in a structured, low-pressure environment outside of the operating theatre without risk for human safety [5,6]. Until now, different methods of simulation have been described, such as training on live animals or cadavers, virtual reality trainers, and video-box trainers. Box trainers are effective for the acquisition of basic laparoscopic skills, shortening operating times, and reducing the risk of perioperative complications [7,8]. However, surgical trainers are not consistently available across all training centers, especially in developing countries where the resources are particularly limited; for this reason, in this setting, the availability of low-cost homemade trainers may represent a noticable advantage [9]. 

The first model of a laparoscopic simulator was described in 1991 by Sackier et al. [10]. After it, several scientific articles about the development and the use of laparoscopic simulators have been published. Li et al. [11] conducted a review of the literature to analyze the differences between commercial and non-commercial laparoscopic simulators and found that although the models of the self-assembly simulators described were simple and affordable options, the main criticisms underscored by the authors were the usual omission of the simulator cost and that a significant part of them had not been routinely subjected to any standardized validation. Since 2014, other studies on laparoscopic boxes have been published, but the critical issues identified by Li et al. have not been satisfactorily resolved [2,12,13,14,15,16,17]. Moreover, to the best of our knowledge, no studies in the literature include clear instructions on how to make a homemade laparoscopic box.

The aim of this study is to provide explicit directions on how to build a simple, low-cost, and realistic homemade box trainer (LABOT). The proposed box trainer has been tested by 25 residents and 35 expert surgeons using, respectively, face validity and content validity questionnaires in order to assess its realism and functional use. Subsequently, 15 students (S group) and 15 residents (R group) completed 3 different tasks (T1, T2, T3) to evaluate the box’s performance during laparoscopic training.

## 2. Materials and Methods 

LABOT has been officially patented. On 09/11/2015, the Ministero dello Sviluppo Economico - Ufficio Italiano Brevetti e Marchi granted its utility model patent (N. 20201500050394).

### 2.1. How to Build LABOT

**Figure 1:**
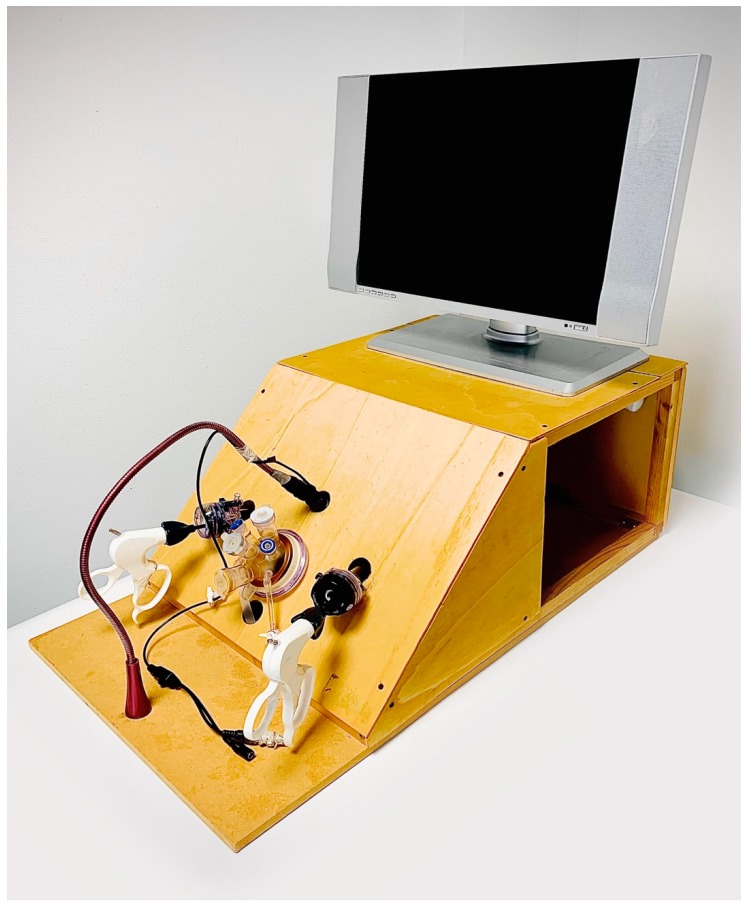
Overview of LABOT.

This low-cost laparoscopic box (Figure 1) is built with the following materials:

- plywood
- medium density fibreboard (MDF)
- rubber (to place the trocar)
- neon lamp (30 cm long)
- bullet mini camera
- low-cost lamp (such as an Ikea lamp, JANSJO^®^)
- screws
- angle Bracket (1 cm large)
- bolt (to fix the camera)
- insulating tape.

All the steps necessary for building this laparoscopic box are explained and illustrated in Appendix A and the Supplementary Figures S1–S6. 

Other figures of LABOT are presented in Supplimentary Figures S7 and S8.

### 2.2. Validation Process

The validation process had the role of defining the potential didactic role of LABOT in:

- simulating the surgical reality;
- having a positive impact on the operator’s learning (learning curve);
- obtaining satisfactory ergonomic results.

As employed previously in the literature [16,17,18,19,20,21], for the validation of the LABOT, we used the following validity models: “face validity,” “content validity,” and “construct validity” [22,23].

**Figure 2:**
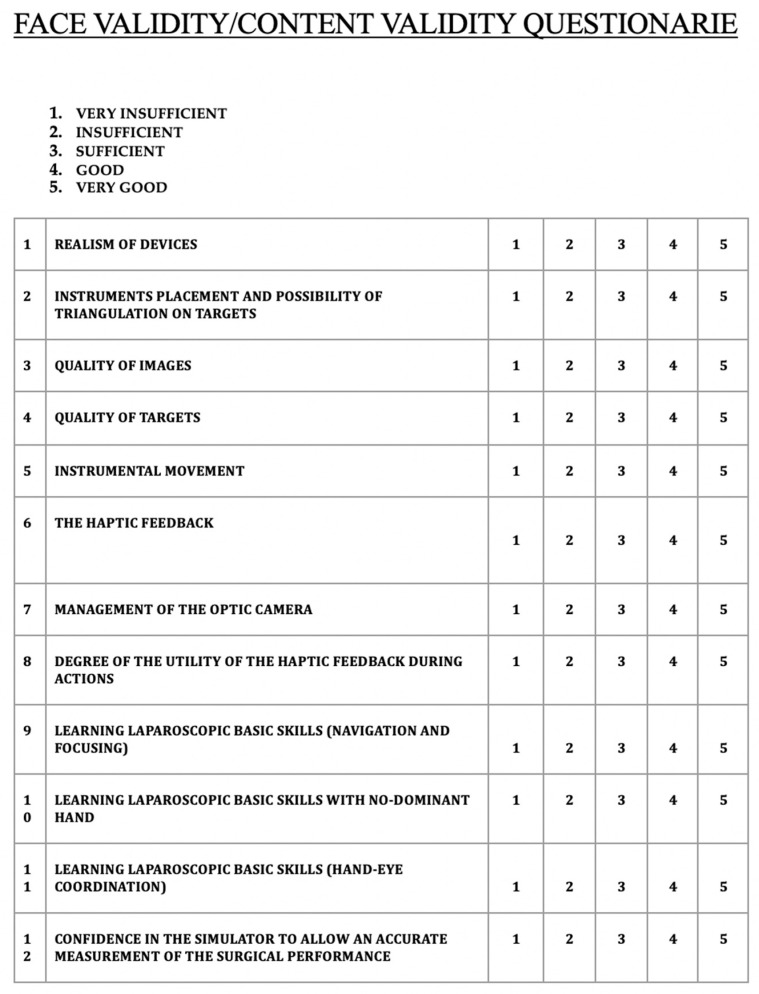
Questionnaire (five-point Likert scale) employed for face and content validities.

Face validity refers to the subjective judgment of the user’s satisfaction with regard to realism of the platform based on personal experience and qualifications. User’s opinions have been collected through a specific closed-ended questionnaire (five-point Likert scale from 1—very insufficient to 5—very good; Figure 2). Overall, 25 residents completed the questionnaire after performing basic and advanced laparoscopic procedures.

Content validity refers to the subjective judgment, only from competent users (in this case, expert surgeons with at least 10 years of experience) about the possibility of learning the proposed techniques by the platform (answering to the question: “Does the simulator really allow for learning the basic skills of laparoscopy?”). Overall, 35 expert surgeons completed the same questionnaire after performing basic and advanced laparoscopic procedures.

Construct validity refers to the ability of the simulator to identify the operators in relation to their actual surgical experience [24,25]. The performance of two groups with different experiences in minimally invasive surgery was compared: 15 students without previous laparoscopic surgical experience (group S) and 15 surgical residents with at maximum 5 years of laparoscopic surgical experience (group R). Three tasks (T1, T2, T3) were performed, during which, the time and the number of procedural errors were evaluated:

- T1 consisted of passing a thread through a ring path; not entering the wire into the ring and an accidental fall of the wire were judged as procedural errors.
- T2 consisted of putting five bolts on top of each other; dropping the bolt was judged a procedural error.
- T3 consisted of passing a bolt from one hand to the other and putting it in a box by using two atraumatic forceps (Joannes type) with the repetition of this procedure five times; dropping the bolt, non-centering, and displacing the box were judged as procedural errors.

All the tasks were repeated half an hour after the end of first set of tasks; the same parameters were evaluated and compared with the previous ones.

The participants performed all the tasks in an isolated room with the lights off in the presence of one expert surgical observer (more than 10 years of experience in teaching laparoscopic procedures) and one experienced trainer surgeon.

This study did not require the approval of the ethics committee because no personal information or clinical news on patients are reported.

### 2.3. Statistical Analysis

The time and number of procedural errors were calculated for each task, making a statistical comparison between the R and S groups. Continuous variables were reported as mean ± standard deviation (±SD) and were compared using the *t*-test; the difference between the two attempts were analyzed using a paired *t*-test. A *p*-value < 0.05 was considered statistically significant. Statistical analysis was performed using SPSS version 20.0 for Windows (SPSS Inc., Chicago, IL, USA) and graphs were made using Excel (Microsoft Corporation, Redmond, WA, USA).

## 3. Results

In both the face and content validity processes, a final score was obtained from all the questionairres administered. Overall, the mean score (±SD) of all answers (approval rating) was 4.18 ± 0.65. Realism of the device was assessed with a mean of 4.25 (±0.60) in face validity and of 4.04 (±0.68) in content validity. There was no statistically significant difference between these two results (*p* = 0.123).

### 3.1. T1 Task

**Figure 3:**
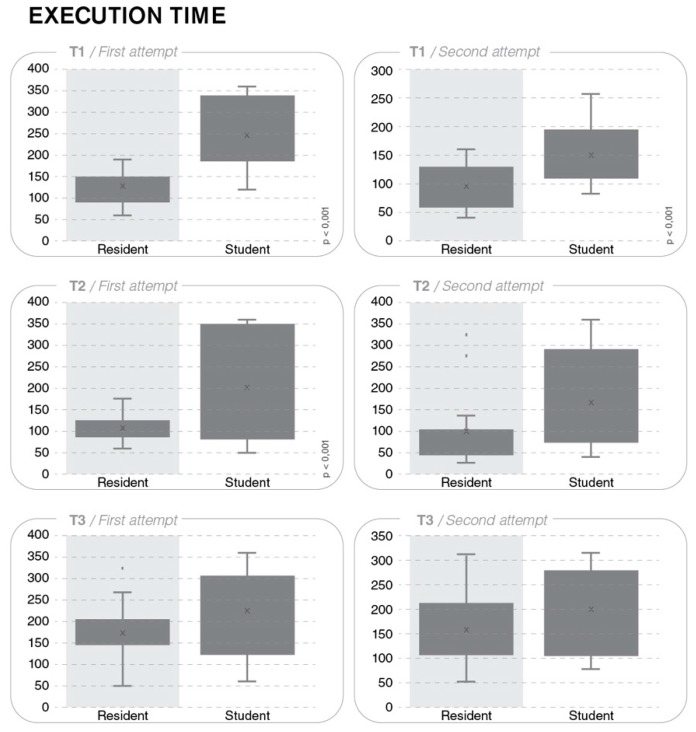
Mean execution time (±SD) for tasks T1–T3.

The mean execution time (±SD) for the first attempt was 248.9 (±84.1) s for the S group and 128.8 (±56.8) s for the R group; for the second attempt the corresponding values were 149.0 (±54.6) s and 97.0 (±41.4) s. For both attempts, the intergroup difference was statistically significant (*p* < 0.001 and *p* = 0.006, respectively) (Figure 3).

**Figure 4:**
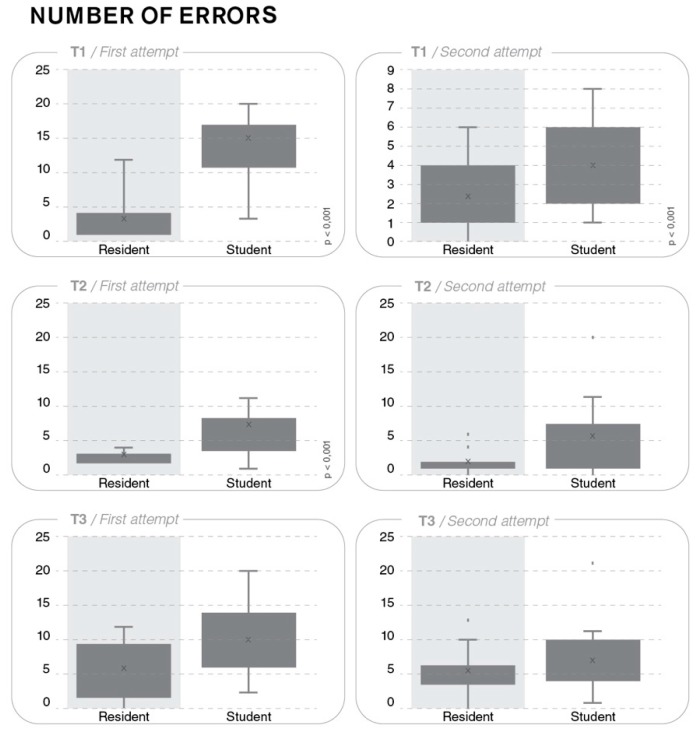
Mean number of procedural errors (±SD) for T1–T3.

The mean number of procedural errors (±SD) was 9.7 (±5.8) for the S group and 2.9 (±1.6) for the R group; for the second attempt, the corresponding values were 3.9 (±2.2) and 2.4 (±1.8). For both attempts, the intergroup difference was statistically significant (*p* < 0.001 and *p* = 0.044, respectively) (Figure 4).

The mean difference for time taken (±SD) between the first and the second attempt was −99.5 (±70.4) s for the S group (*p* < 0.001) and −31.3 (±43.1) s for the R group (*p* = 0.014). The mean differential number of errors was −5.7 (±5.1) for the S group (*p* = 0.001) and −0.5 (±1.6) for the R group (*p* = 0.229).

### 3.2. T2 Task

The mean execution time for the first attempt was 200.3 (±123.4) s for the S group and 105.6 (±32.4) s for the R group; for the second attempt, the corresponding values were 170.1 (±119.2) s and 99.1 (±88.1) s. The intergroup difference was statistically significant for the first attempt (*p* = 0.008), but not for the second attempt (*p* = 0.074) (Figure 3).

The mean number of procedural errors was 7.1 (±5.8) for the S group and 2.8 (±0.8) for the R group; for the second attempt, the corresponding values were 5.7 (±6.6) and 1.8 (±1.7). For both attempts, the intergroup difference was statistically significant (*p* = 0.008 and *p* = 0.032, respectively) (Figure 4).

The mean difference in time between the first and the second attempt was −30.1 (±141.3) s for the S group (*p* = 0.423) and −6.5 (±74.7) s for the R group (*p* = 0.742). The mean differential number of errors between the first and the second attempt was −1.4 (±9.0) for the S group (*p* = 0.558) and −1.0 (±1.8) for the R group (*p* = 0.246).

### 3.3. T3 Task

The mean execution time for the first attempt was 229.0 (±93.8) s for the S group and 178.9 (±80.3) s for the R group; for the second attempt, the corresponding values were 199.3 (±85.6) s and 155.8 (±68.1) s. The intergroup difference was not significant for the first (*p* = 0.127) and the second attempts (*p* = 0.136) (Figure 3).

The mean number of procedural errors (± SD) was 10.3 (±5.2) for the S group and 5.8 (±3.7) for the R group; for the second attempt, the corresponding values were 7.1 (±4.8) and 4.8 (±3.3). The intergroup difference was statistically significant for the first attempt (*p* = 0.011), but not for the second attempt (*p* = 0.146) (Figure 4).

The mean difference in time between the first and the second attempt was −29.7 (±99.5) s for the S group (*p* = 0.423) and −23.1 (±67.8) s for the R group (*p* = 0.209). The mean differential number of errors between the first and the second attempt was −3.3 (±5.9) for the S group (*p* = 0.050) and −1.0 (±2.7) for the R group (*p* = 0.173).

## 4. Discussion

**Figure 5:**
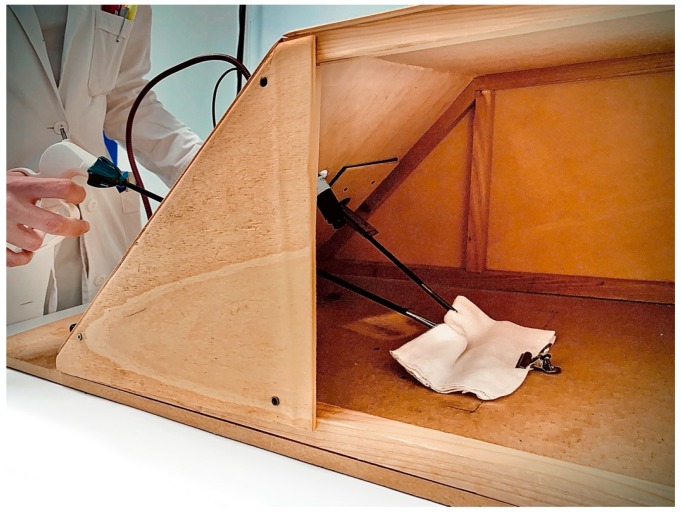
Internal operative space of LABOT during the performance of an easy training exercise.

LABOT is a low-cost laparoscopic box that can be easily home-built by using readily available materials. It has a resistant, stable, and ecological durable structure. Although it is easily transportable, this box has a large internal operative space (Figure 5).

Cost is a limiting factor for the accessibility of simulator models; in fact, about 46% of non-commercial simulators provide a figure for cost [11]. The cost of current available training boxes ranges from £3 to £216; the cost of our box is £68 (around 80 euros; this amount refers to the cost of materials and assembly).

In the literature, the most common material used to simulate the abdominal wall and cavity is plastic (Table 1) [14,24,25,26,27,28,29,30,31,32]. For simulating the abdominal wall, we decided to build LABOT by using plywood and rubber, which are cheap and easy to manipulate. The actual shape of LABOT allows for a wide surgical view, a good instrument triangulation, and an optimal fixing of the camera with 30° vision of the operative field. We employed a neon lamp as a light source, different from around 10% of the other authors, who preferred using an LED lamp [11]; in fact, we deemed that LED lamps are small but very complex to assemble, as well as being fragile, whereas neon lamps are more accessible, economical, resistant, and easy to mount on the box. Moreover, neon lamps have a multidirectional light source (360°), which permits the creation of a wide cone of light influence, allowing for an adequate brightness. In contrast, LED lamps are limited by an omnidirectional light source, which causes a lower brightness. Li et al. reported the use of a webcam or a laparoscope as visualization systems for the box (Table 1) [11]. We have tested about 10 devices (webcam, endoscope camera, bullet camera, phone connected camera) with the following observations: the webcams allowed for autofocus, but the image, even if at high definition, tended to be grainy and the vision of the movement was not fluid and often delayed; in addition the webcams usually needed to be connected to a laptop. The low-cost endoscopic cameras, despite having an auto-lighting system, had a very low definition, and having a digital signal, shared some of the disadvantages of webcams. Some authors used tablets as both the camera and monitor; however, they required the construction of a rigid support and have an operative visualization related to a small screen. For all these reasons, we decided to use a bullet mini camera fixed on a flexible support for the LABOT. Thanks to its cylindrical shape, it allows the users to get a closer look in the operative field and to turn the image; moreover, it can be manipulated almost like a laparoscope and connected to any television set because of the analogic output, allowing for a fluid vision of movements. However, the bullet camera needs a manual focus.

We deemed that in laparoscopic boxes, the visualization using PC monitor or TV screen represents an inexpensive solution. In LABOT, the camera monitor is represented by a TV screen, on which we did not adopt any particular image settings. Some authors reported the use of non-electronic methods of visualization, such as mirrors and direct vision, but these options appeared unrealistic and were characterized by low-quality outcomes [16,31,33,34,35]. Overall, the final video quality depended on multiple factos, such as the camera, image zoom, and intensity of the light source.

Li et al. reported that about the half of the home-made boxes have not been reliably validated and tested in surgical tasks (Table 1) [11]. Some studies compared home-box trainers and commercial trainers to understand whether home-box trainers can represent a low-cost solution for improving a surgeon’s abilities [2,9]. Couto et al. accurately described their low-cost laparoscopic box but they did not apply any tests of performance [13]. Other authors have tested their home-made boxes with different modalities: Oti et al. performed exercises similar to ours, obtaining similar positive results, but the authors tested their box by enrolling only 20 students without surgical experience [14]. Thinggaard et al. recruited surgeons and trainers (60 subjects) to validate their box with different exercises [15]. Aslam et al. assessed their simulator with 34 surgical trainers, giving instructions to build the box, and finally analyzing the cost, time of construction, and realization of simple exercises [14].

Some studies have evaluted the positive impact on the learning surgical curve of laparoscopic trainers in developing countries, where it is difficult to obtain expensive instruments and innovative materials [36,37,38]. Thanks to the low cost and accessibility of materials, LABOT could represent a suitable solution in these countries.

We designed this study in order to understand whether LABOT was potentially suitable for surgical training. We selected three tasks with increasing difficulty in order to assess oculo-motor coordination, the ability to manipulate objects, the subtlety of movements, and the perception of three-dimensionality. The validation process on movements and the possibility of triangulation of the laparoscopic instruments indicated that LABOT may be suitable for learning adequate laparoscopic movements. The haptic feedback seemed to be well represented; in fact, both resident and surgeon groups specifically attributed to LABOT the capability to improve laparoscopic hand-eye coordination. The most relevant issue for this laparoscopic box seemed to be related to image quality and camera support; in fact, lower quality results were obtained for specific questions about these aspects. Overall, by comparing results of the face and content validity process, it appears that residents and surgeons agreed in identifying the same advantages and disadvantages of LABOT (*p* = 0.123). However, it must be noted that the results of the face and content validity processes are limited in the current literature by the absence of validated and standardized questionnaires, by which comparison with other tools for surgical training should be done.

We considered a proof of the learning curve to be the difference between the results of the second and the first attempt of the operative tasks. The data analysis confirmed that operators with more experience in laparoscopic surgery tended to have better procedural outcomes. In particular, these reports found a statistical confirmation for the simplest surgical tasks (T1 and T2). On the contrary, a more complex exercise (T3) had a longer learning curve for both groups. Therefore, in the initial phase of the training time, the number of procedural errors tended to be similar between the two groups; however, the S group had the tendency to have greater learning improvements than the R group. This data seems to suggest that LABOT may be particularly useful in the initial phase of laparoscopic learning.

At the moment, we are testing LABOT on more complex procedures, such as the execution of intracorporeal nodes and complete simulated surgical operations (i.e., appendicectomy). However, we hypothesize that the lack of a second operator to steer the camera (that is not expected by using this box) may make the execution of these tasks more difficult. In the near future, we are going to analyze and present this data, aiming to draw a conclusion about the role of LABOT on advanced surgical skills.

## 5. Conclusions

LABOT is a low-cost laparoscopic simulator, whose instructions provided in this manuscript allows for its easy construction at home. This box permits improvement of basic technical skills, especially at the beginning of surgical training. In the future, further studies should evaluate the potential of this laparoscopic box for long-term surgical training with more complex tasks, attempts of simulation, and validated paths of laparoscopic skill learning.

## Figures and Tables

**Table 1:** Analysis of the characteristics of some of simulator boxes published in the recent literature.

Undergone Validation	Yes (43%)	No (57%)			
Figure for cost	Yes (46%)	No (54%)			
Abdominal Wall	Plastic (52%)	Cardboard (16%)	Plywood (5%)	Others (27%)	
Light Source	External light (36%)	Laparoscope (16%)	Desk lapm (12.5%)	LED (7%)	Others (28.5%)
Visualization	TV o PC screen Phone and digital Camera (61%)	Laparoscope (23%)	Direct vision (3.5%)	Mirror (3.5%)	Others (9%)
Monitor	PC, tablet, laptop (34%)	TV screen (25%)	Video monitor (16%)	Other (25%)	

Data were extracted via analysis of the following references [9,11,12,13,24,25].

## Supplementary Materials

The following are available online at https://www.mdpi.com/1660-4601/17/1/323/s1, Figure S1: Instructions for Building LABOT—Part 1, Figure S2: Instructions for Building LABOT—Part 2, Figure S3: Instructions for Building LABOT—Part 3, Figure S4: Instructions for Building LABOT—Part 4, Figure S5: Instructions for Building LABOT—Part 5, Figure S6: Lateral view of LABOT after insertion of the laparoscopic intruments in trocars, Figure S7: Internal view of LABOT after insertion of the laparoscopic intruments in trocars. Figure S8: Internal view of LABOT.

## Appendix A

### Instructions for Building LABOT

Supplementary Figure S1: Wooden materials and relative fastening means necessary for the construction of LABOT (all measurements, such as length, height, and thickness, are reported in the figure). The box (base 1.5 cm × 1.5 cm) is composed of an inner framework characterized by wooden poles and an external cover made of plywood with a thickness of 0.3 cm. Panels A and B need holes with diameter of 2 cm, which can be easily done using a drill. In panel A (frontal panel, 40.6 × 35.3 cm), two holes must be positioned on the median line at a distance of 20 cm from each other and one hole at 5 cm up the midline. In panel B, only a central hole needs to be drilled. The trocars are positioned in the two holes on the midline (panel A) and the camera in the central hole (panel B).

Supplementary Figure S2: The subsection I shows the flexible support allowing for the introduction of the trocars; it is constituted by two 8-cm rubber floor squares, which have two small holes in their center. The subsection II describes the electronic materials necessary for the construction of LABOT; in particular, the low-cost lamp must be handily modified (see Supplementary Figure S3). The subsection III describes the base of the box, which is made of MDF (medium density fiberboard). In the frontal side of the base, two holes must be drilled in order to fix the camera support (the modified low-cost lamp). These holes must be done after having disassembled the low-cost lamp (see Supplementary Figure S5).

Supplementary Figure S3: In subsection I, four wooden poles (2 × 40-cm units; 2 × 51-cm units) are fixed with screws on the four sides of the MDF base. Subsequently, two wooden poles (2 × 22-cm units) must be fixed using the aluminum bundles and the screws, as shown in the subsection II, 27.5 cm apart from each other. Then, chamfer poles (1 × 40-cm, 1 × 22-cm, and 1 × 33-cm units) must be fixed, as shown in the figure.

Supplementary Figure S4: In the frontal part of panel A, the three panels (A, B, C) must be joined with screws; in particular, the rubber panel (C) must be positioned between the others two (A and B).

Supplementary Figure S5: In the subsection I, the light is fixed on panel D. A 32-cm wooden strip must be inserted between the light and this panel. In subsection II, the instructions for building the bulled camera support by modifying the low-cost lamp are shown: the head of the lamp must be cut with a hacksaw, as shown at point A; the lamp base must be removed by unscrewing the two screws, as shown at point B; a bolt must be fixed on the head of the lamp using adhesive tape, as shown at point D; then, the bolt must be fixed at the bottom of bullet camera.
