# Supplementary figures and images for: Development and Validation of a Homemade, Low-Cost Laparoscopic Simulator for Resident Surgeons (LABOT)
Source: Int J Environ Res Public Health. 2020 Jan 2;17(1):323. doi: 10.3390/ijerph17010323 (PMC6981870; doi:10.3390/ijerph17010323)

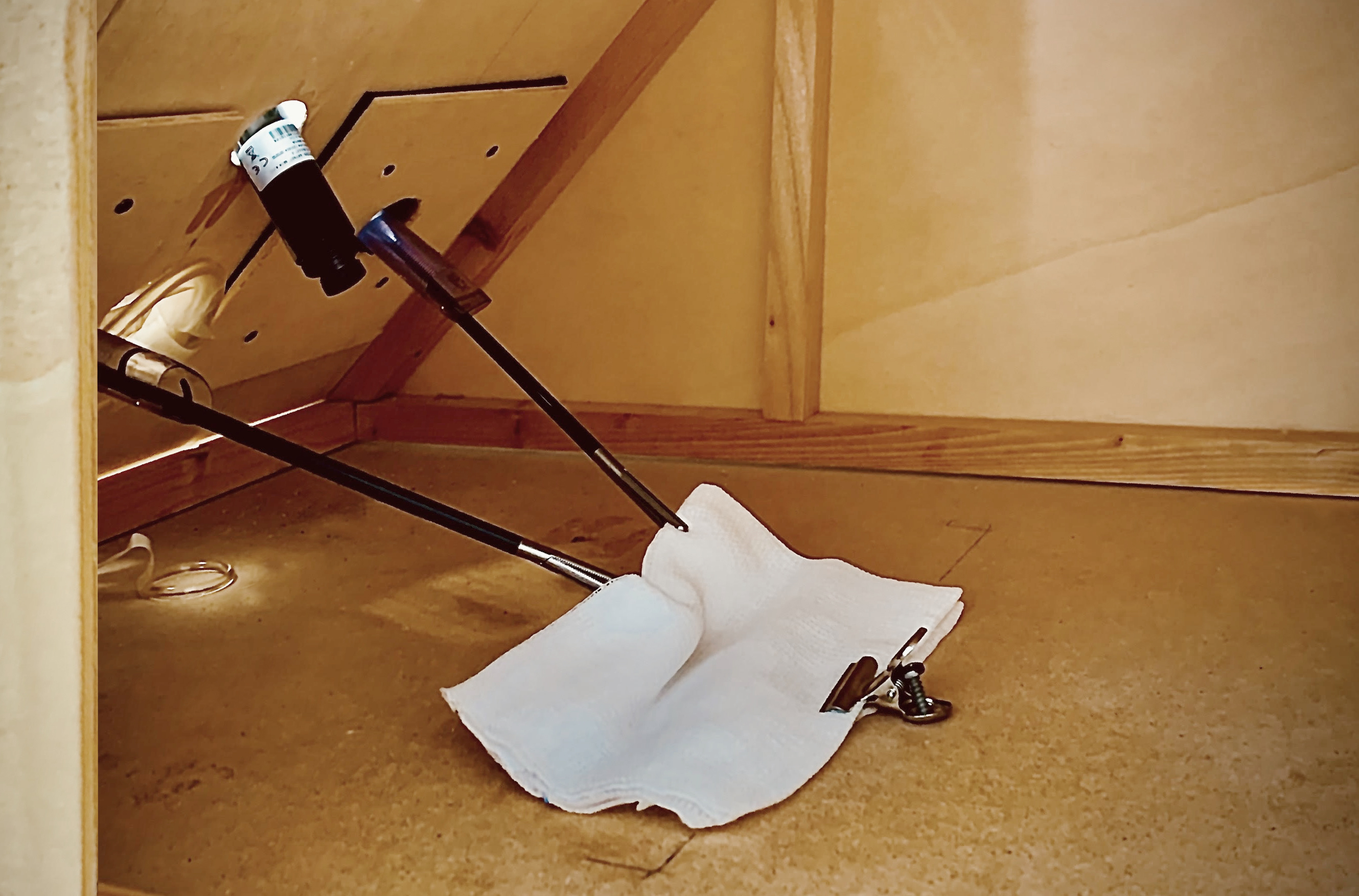

Supplement: Supplementary file 1 [file ijerph-17-00323-s001.zip › Supplementary figures LABOT/Supplementary Figure 8.jpg]

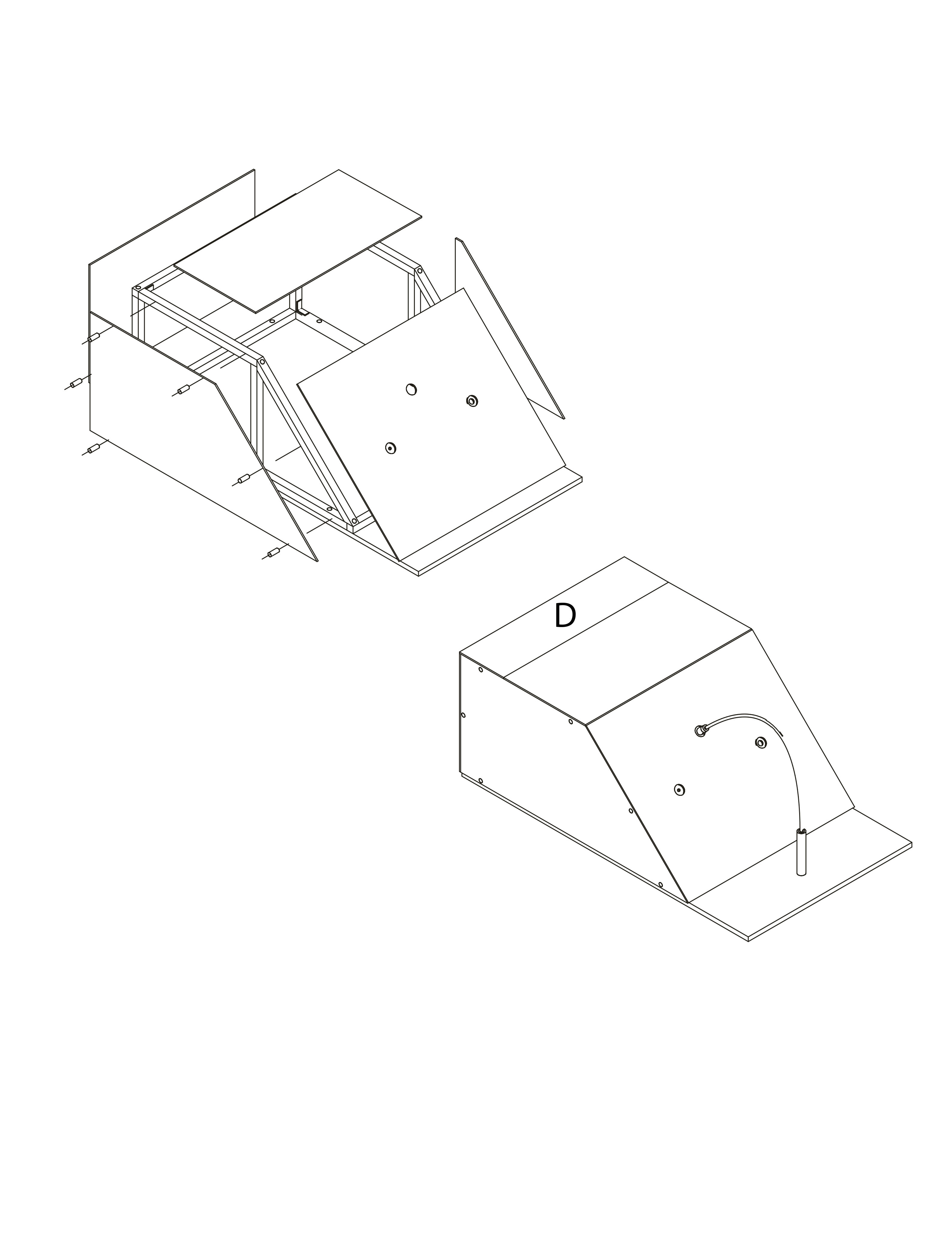

Supplement: Supplementary file 1 [file ijerph-17-00323-s001.zip › Supplementary figures LABOT/Supplementary Figure 6.jpg]

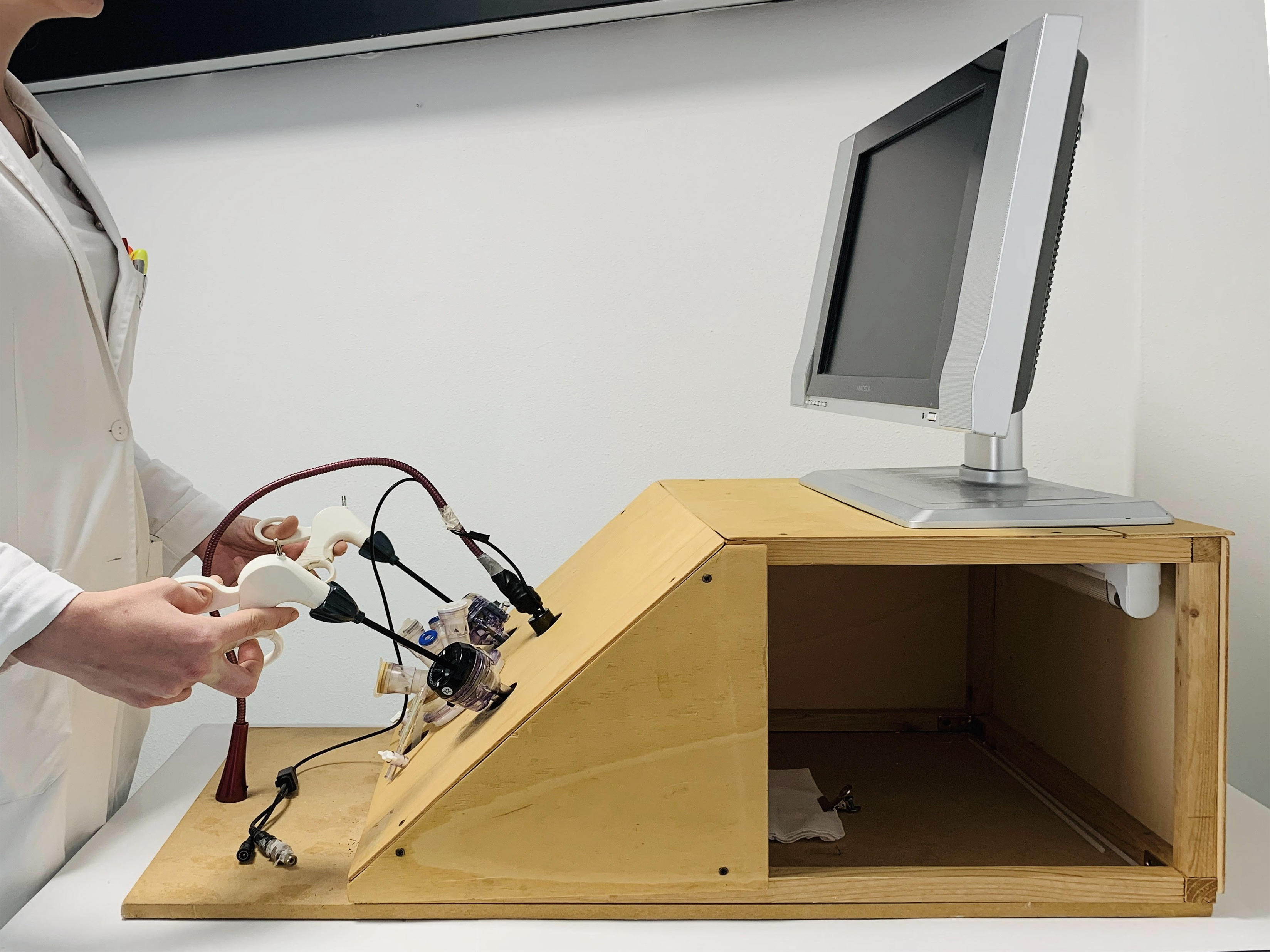

Supplement: Supplementary file 1 [file ijerph-17-00323-s001.zip › Supplementary figures LABOT/Supplementary figure 7.jpg]

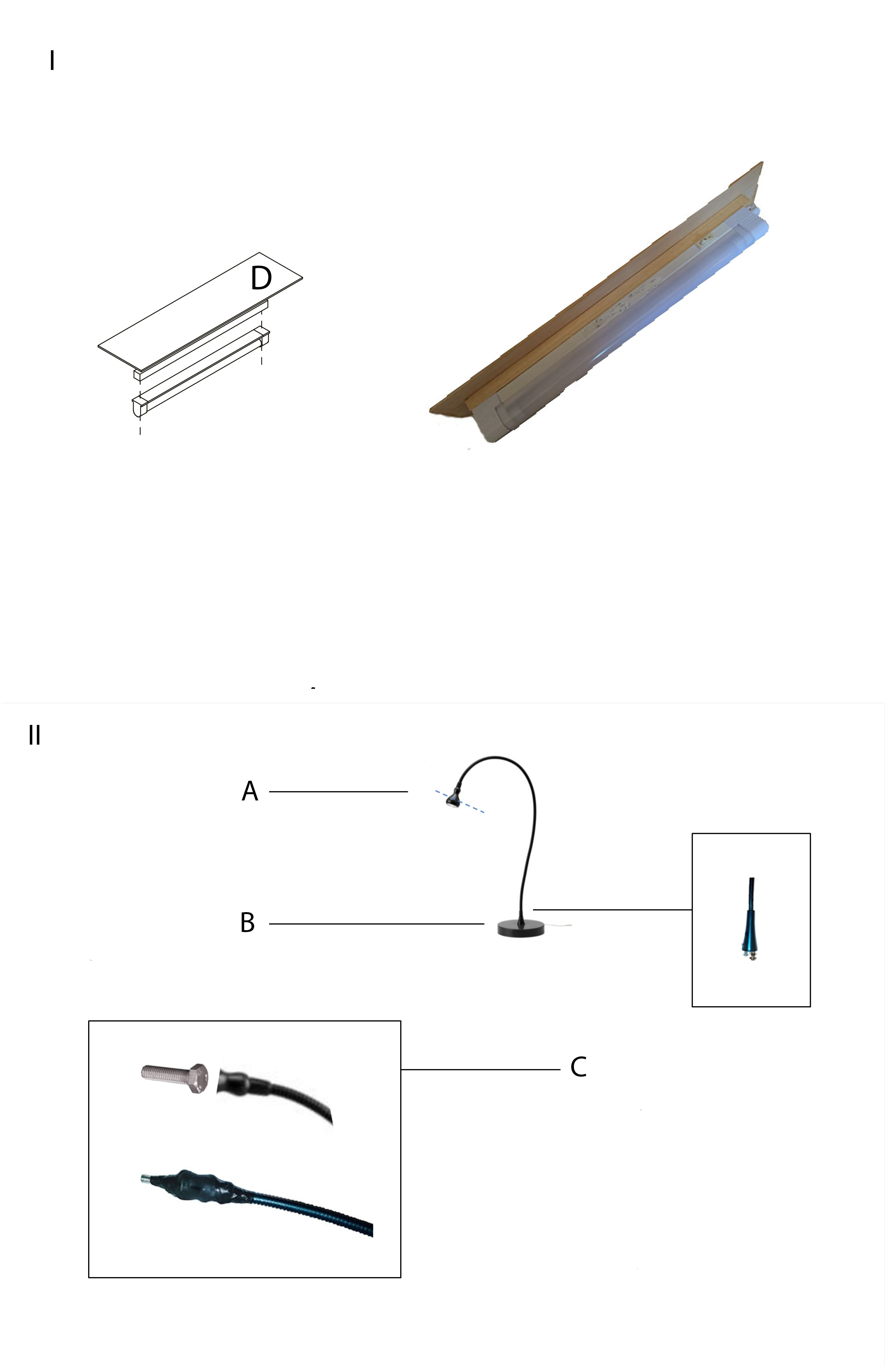

Supplement: Supplementary file 1 [file ijerph-17-00323-s001.zip › Supplementary figures LABOT/Supplementary figure 5.jpg]

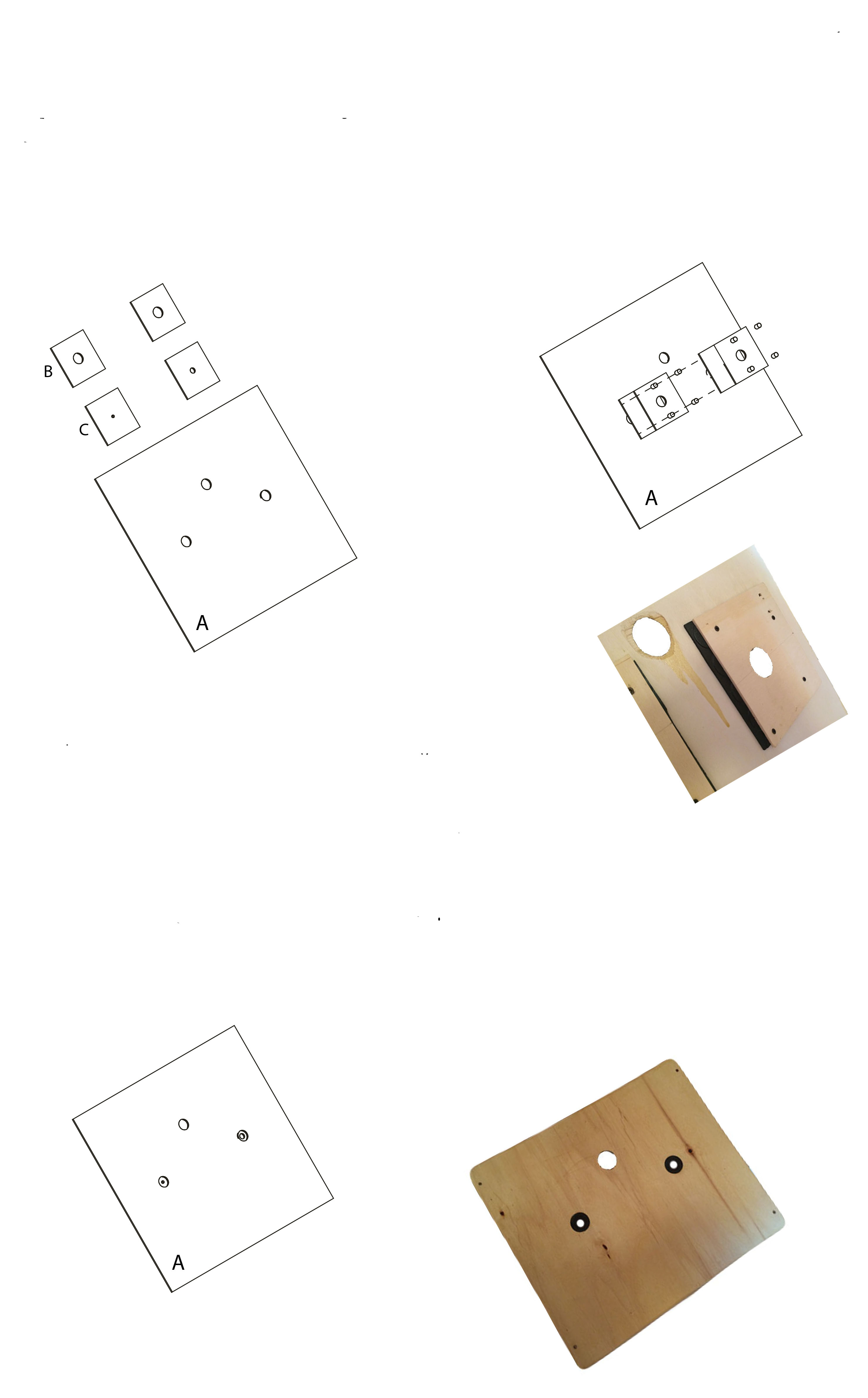

Supplement: Supplementary file 1 [file ijerph-17-00323-s001.zip › Supplementary figures LABOT/Supplementary figure 4.jpg]

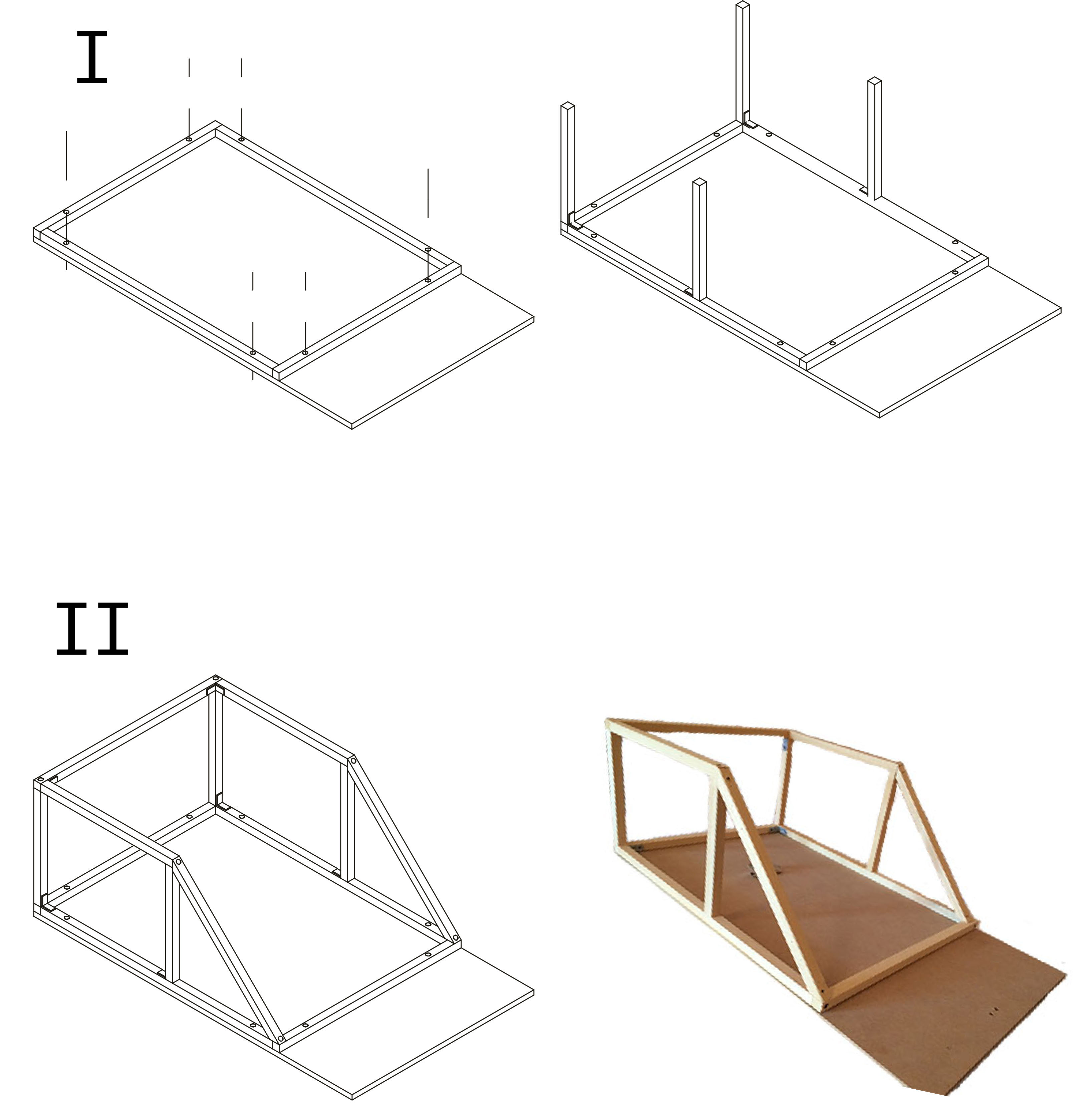

Supplement: Supplementary file 1 [file ijerph-17-00323-s001.zip › Supplementary figures LABOT/Supplementary figure 3.jpg]

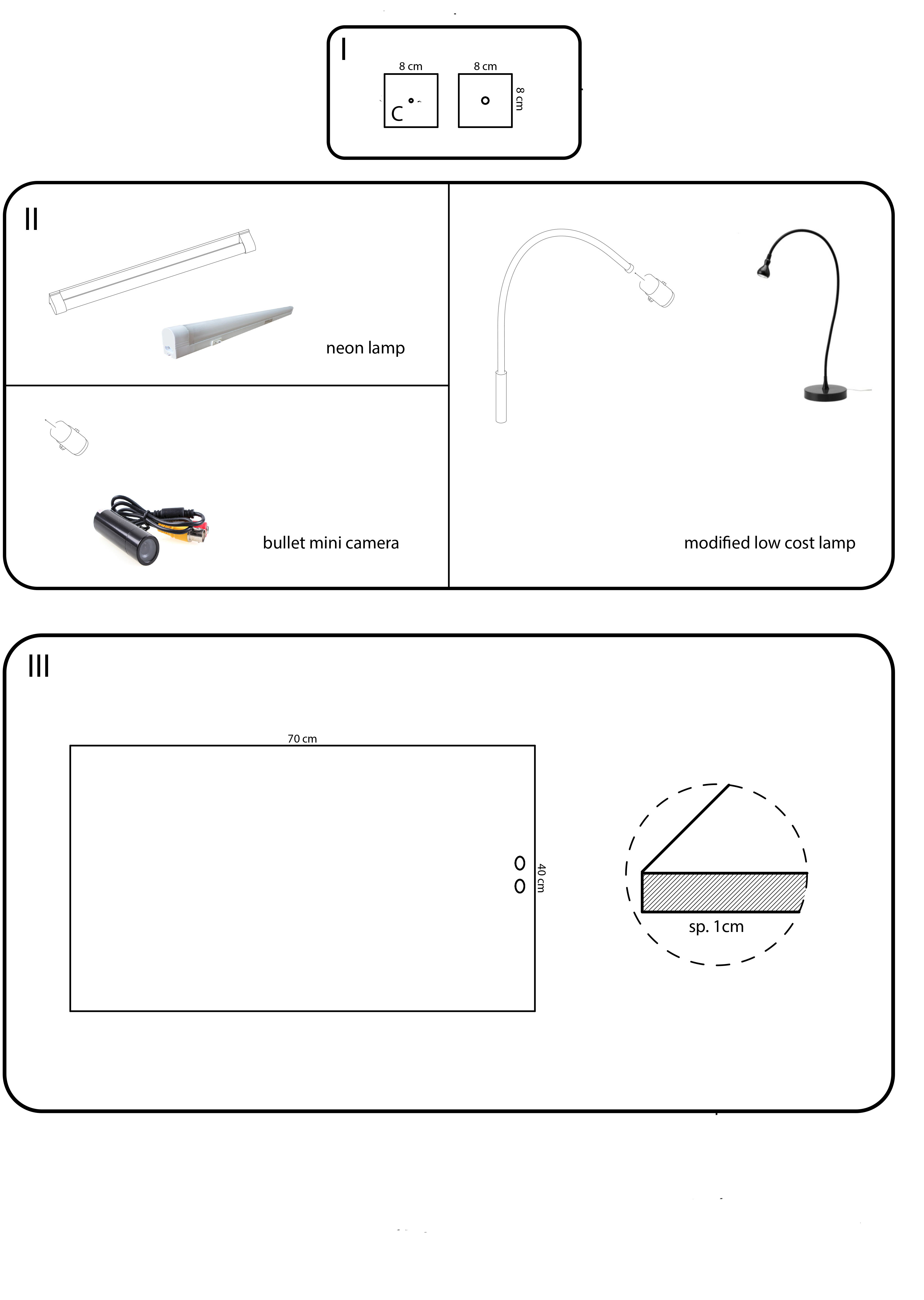

Supplement: Supplementary file 1 [file ijerph-17-00323-s001.zip › Supplementary figures LABOT/Supplementary figure 2.jpg]

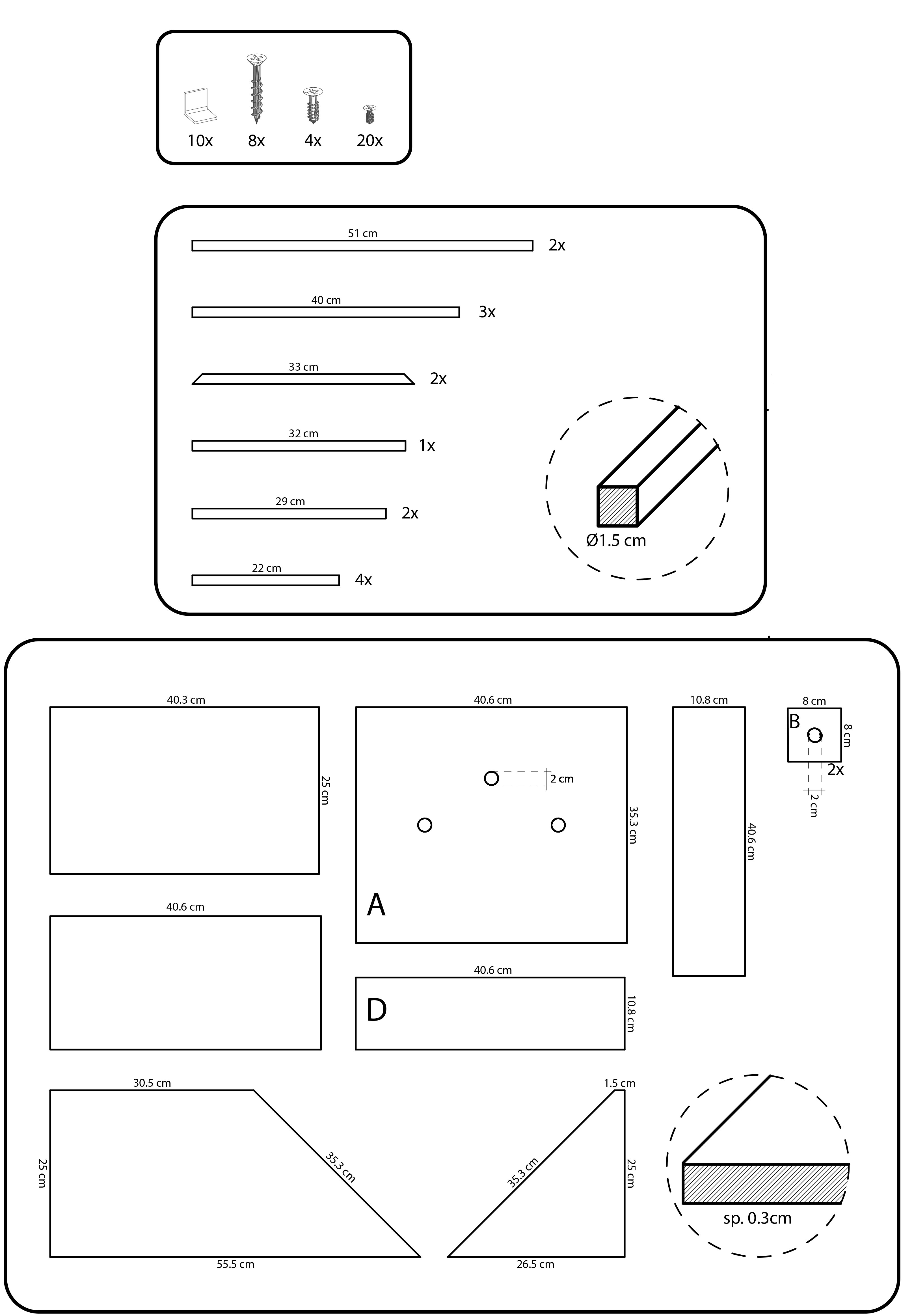

Supplement: Supplementary file 1 [file ijerph-17-00323-s001.zip › Supplementary figures LABOT/Supplementary figure 1]
